# Supplementary figures and images for: Phosphoproteomic insights into the regulation of root length in rice (Oryza sativa L. cv. KDML 105): uncovering key events and pathways involving phosphorylated proteins
Source: PeerJ. 2025 Jul 4;13:e19361. doi: 10.7717/peerj.19361 (PMC12232931; doi:10.7717/peerj.19361)

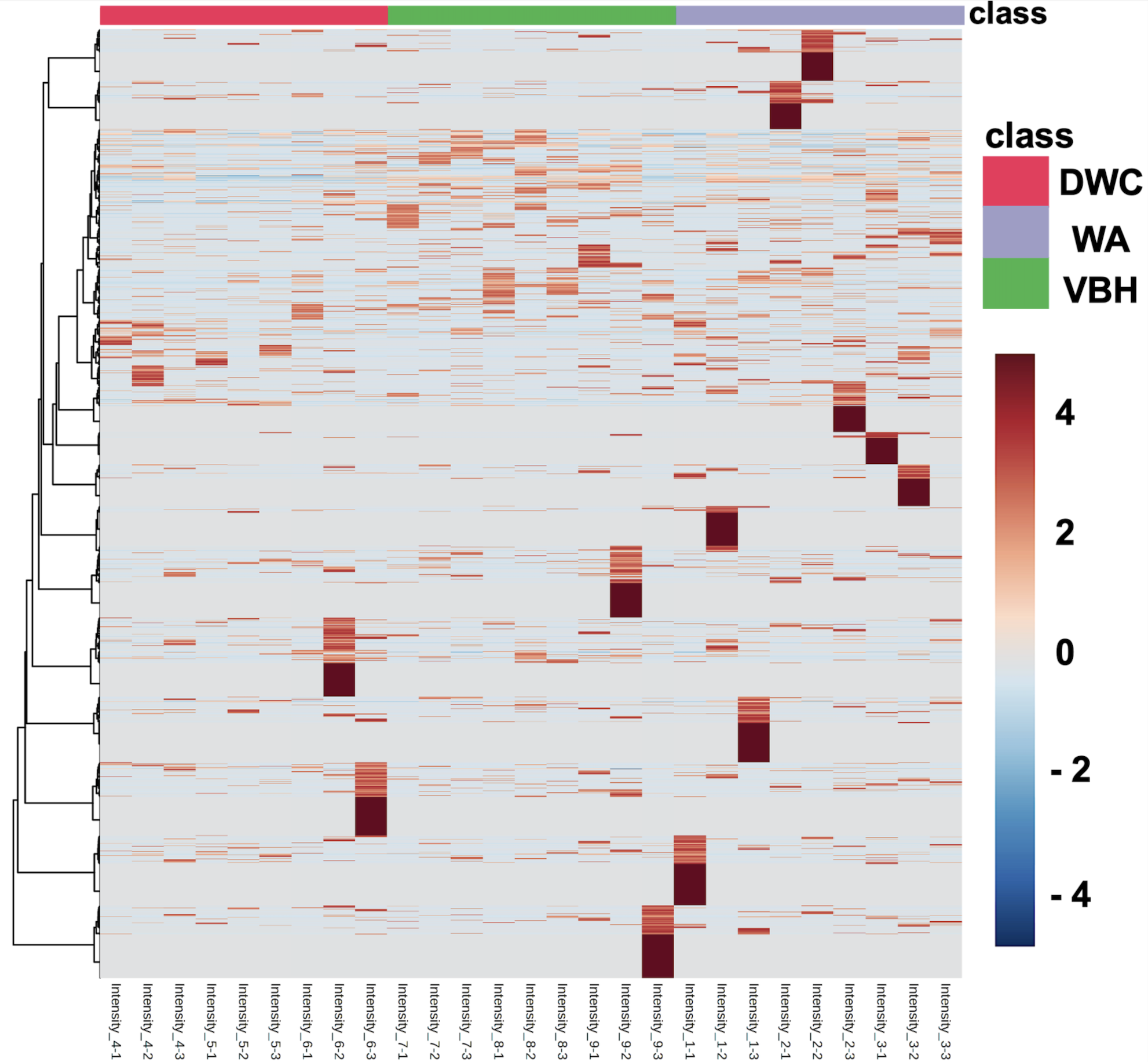

Supplement: Supplemental Information 1 [file peerj-13-19361-s001.png]
